# Case Report: Multifactorial weaning failure after lung transplantation in paraquat-induced pulmonary fibrosis: a case-based clinical review

**DOI:** 10.3389/fmed.2025.1725363

**Published:** 2026-01-21

**Authors:** Xin Xia, Yue Xing, Tianjun Zhou, Xiaosong Ben, Weifeng Zhan

**Affiliations:** 1 Department of Thoracic Surgery, Guangdong Provincial People's Hospital (Guangdong Academy of Medical Sciences), Southern Medical University, Guangzhou, China; 2 Department of Organ Transplantation, Guangdong Provincial People's Hospital (Guangdong Academy of Medical Sciences), Southern Medical University, Guangzhou, China

**Keywords:** lung transplantation, paraquat poisoning, post-operative rehabilitation, toxic lung injury, ventilator weaning

## Abstract

**Introduction:**

Lung transplantation (LT) remains a rare but life-saving option for end-stage pulmonary fibrosis secondary to paraquat poisoning. However, the post-operative course can be complicated by prolonged ventilator dependence, particularly in patients with toxic lung injury who have received extended sedation and neuromuscular blockade.

**Case report:**

A 25-year-old male presented with acute paraquat intoxication and developed progressive respiratory failure despite standard decontamination and hemoperfusion. He underwent bilateral LT after bridging with extracorporeal membrane oxygenation (ECMO). Following surgery, although gas exchange was satisfactory, repeated extubation attempts failed due to respiratory muscle weakness. Through a structured, multidisciplinary protocol combining serial neuromuscular assessment, individualized rehabilitation, and gradual ventilator weaning, successful extubation was achieved on post-operative day 12. The patient was discharged on day 48 with stable graft function. A review of 15 previously reported paraquat-related LT cases revealed recurring challenges in transplant timing, ECMO bridging, and respiratory recovery.

**Conclusion:**

This case highlights that in paraquat-induced toxic lung injury, successful ventilator liberation after LT depends on early recognition of neuromuscular dysfunction and integration of targeted rehabilitation within a multimodal, individualized care framework rather than relying solely on oxygenation parameters.

## Case presentation

**Figure 1:**
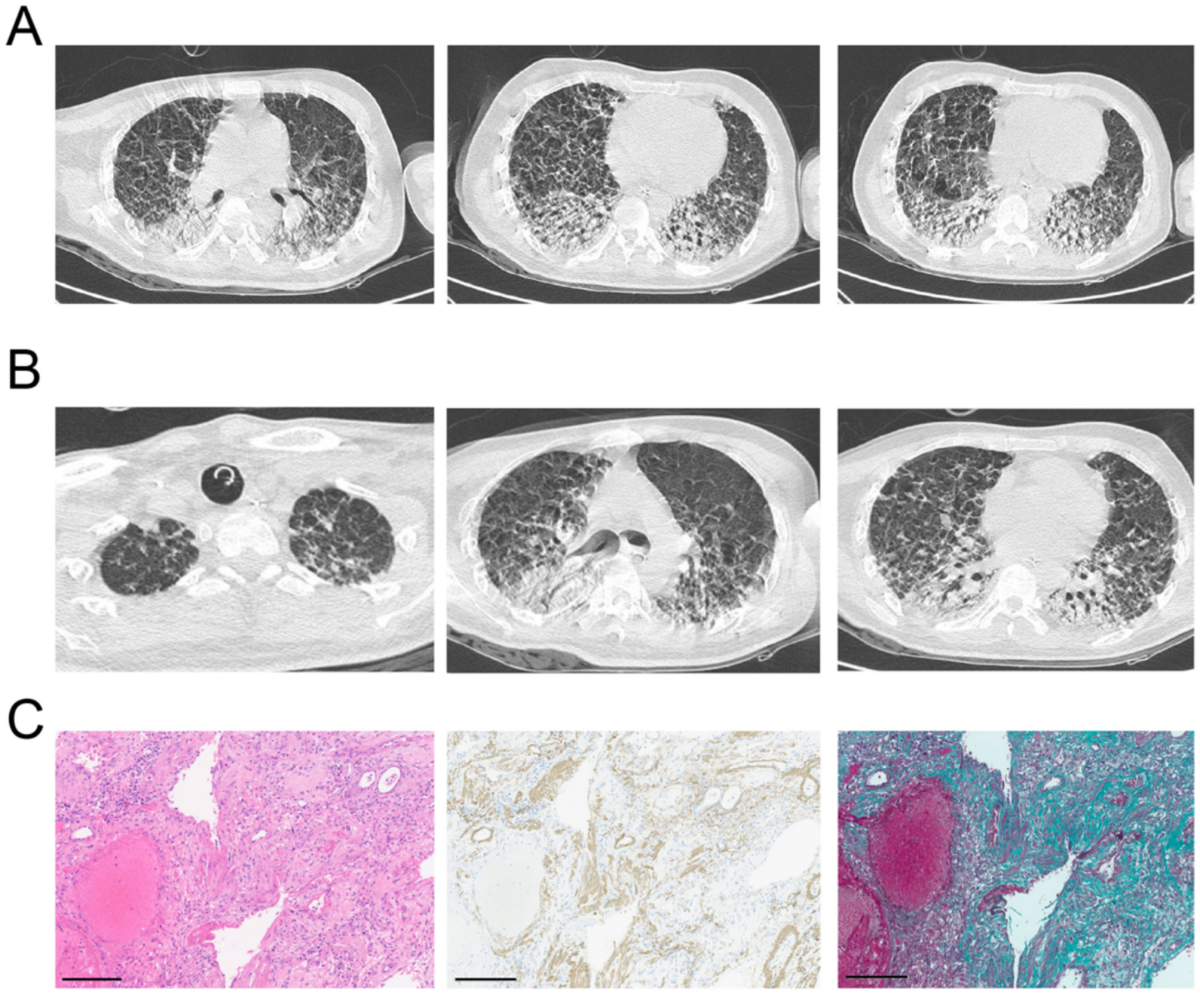
Chest CT evolution before lung transplantation. **(A)** Chest CT scan on Day 5 post-paraquat ingestion shows early bilateral ground-glass opacities with subpleural predominance. **(B)** Radiologic progression to extensive consolidation and fibrotic remodeling, consistent with irreversible pulmonary damage. **(C)** Histopathologic analysis of the explanted native lung reveals diffuse fibrosis and fibroblast proliferation.

A 25-year-old male from Zhaoqing, Guangdong Province, China, with a background of recent psychological stress and two prior episodes of self-harm, ingested approximately 20 mL of 20% paraquat solution on August 16, 2023, in a suicide attempt. He initially presented with severe nausea and vomiting, and was treated at a local hospital with

gastric lavage, catharsis, hemoperfusion, and other supportive measures. However, by day 5 post-ingestion, the patient developed progressive dyspnea, and chest computed tomography (CT) revealed extensive bilateral pulmonary infiltrates with exudation and consolidation (Figure 1A). By August 23, his blood paraquat concentration was 99.15 μg/L, and his respiratory status deteriorated further, necessitating tracheal intubation and invasive mechanical ventilation. A diagnosis of paraquat poisoning with evolving pulmonary fibrosis was established.

On September 14 (day 29), the patient was transferred to our center due to worsening hypoxemia despite maximal ventilatory support (FiO_2_ 1.0, PaO_2_ 54 mmHg, PaCO_2_ 75 mmHg, pH 7.15). Imaging demonstrated progression of pulmonary fibrosis with bilateral pleural effusions and pneumothorax (Figure 1B). High inflammatory markers (procalcitonin 4.13 ng/mL; IL-6 193 pg/mL) and positive cultures (Sputum cultures yielded *Klebsiella pneumoniae*, and blood cultures were positive for *Staphylococcus spp*.) indicated superimposed infection. The patient was started on veno-venous extracorporeal membrane oxygenation (VV-ECMO) via right internal jugular to femoral vein cannulation (gas flow 2 L/min, blood flow 3.68 L/min, RPM 3050). Heart and kidney functions were preserved, liver function was mildly impaired but improved with hepatoprotective therapy, and coagulation remained stable. The paraquat level and liver and kidney function markers were provided in our Supplementary Figure 1.

On day 34 post-ingestion (September 19), the patient underwent sequential bilateral lung transplantation under continued ECMO support. The donor was a 42-year-old male who died of stroke. Intraoperative findings showed no pleural adhesions. Cold ischemia times were 5.5 and 11 h for the right and left lungs, respectively. The surgery lasted 10 h with estimated blood loss of 600 ml. The patient was transferred to the ICU and ECMO was successfully weaned off on post-operative day (POD) 1. Final pathology of the explanted lungs revealed dense interstitial fibrosis with hemorrhage and immune staining for smooth muscle actin (+++), confirming paraquat-induced fibrotic injury (Figure 1C).

**Figure 2:**
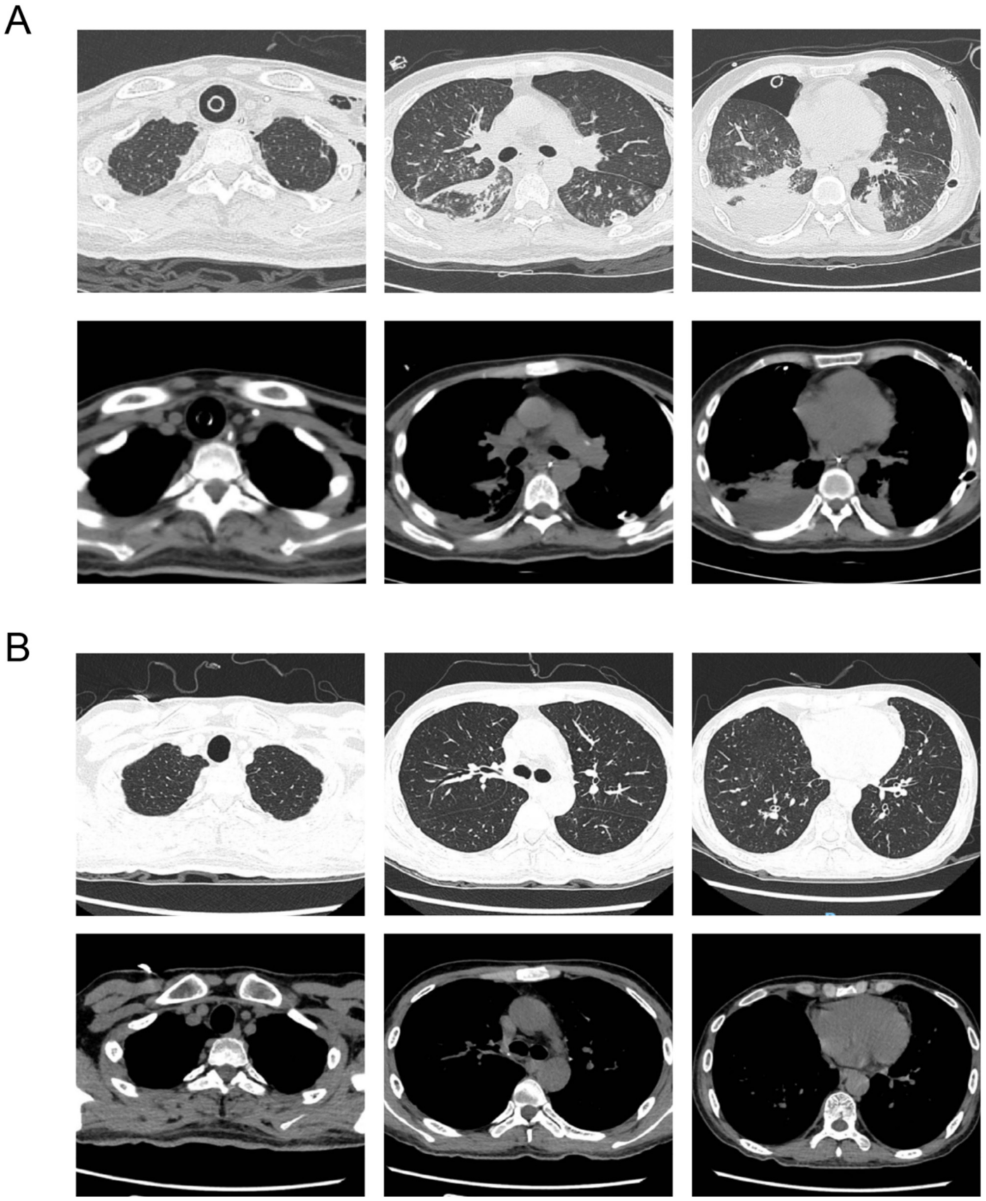
Chest CT evolution after lung transplantation. **(A)** Chest CT obtained one week after lung transplantation shows clear donor lungs with no signs of infection or rejection. **(B)** Follow-up CT at 1 month post-transplant demonstrates stable graft appearance and improved aeration, with no radiologic complications.

Post-operative management included broad-spectrum antimicrobial therapy (ceftazidime–avibactam, vancomycin, voriconazole) and triple immunosuppression (tacrolimus, prednisone, and mycophenolate mofetil). BAL cultures grew multidrug-resistant *Acinetobacter baumannii*. No acute rejection was observed (Figure 2A).

**Figure 3:**
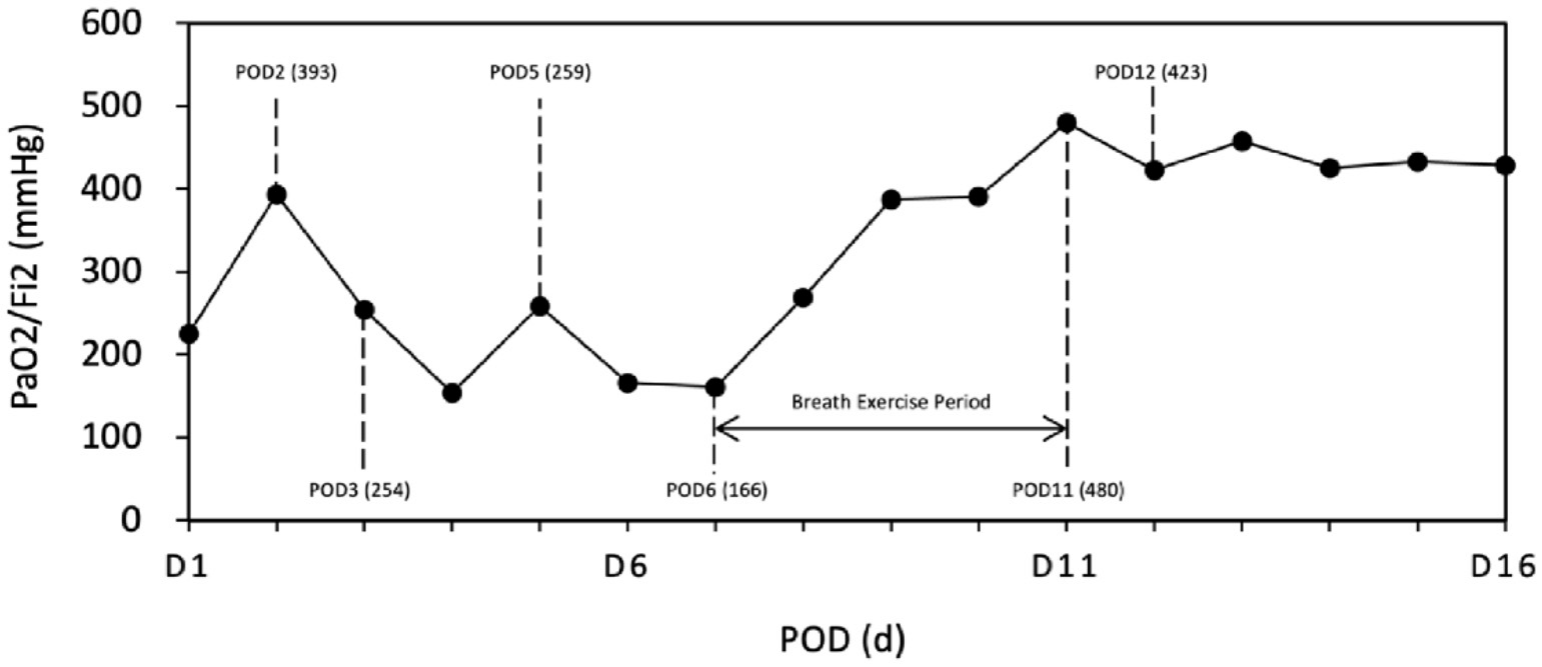
Oxygenation index (PaO_2_/FiO_2_) from surgery to the 16th post-operative day (POD). POD 1, ECMO removed; POD 2, Successful Spontaneous Breathing Trial (SBT), indicating safe removal of MV; POD 3, Ventilator reintroduced due to a significant drop in oxygenation (PaO_2_/FiO_2_: 254); POD 5, Second extubation attempt unsuccessful (PaO_2_/FiO_2_: 259); POD 6–11, Active implementation of respiratory strengthening exercises (POD 6–POD 11); POD 12, Successful third extubation attempt (PaO_2_/FiO_2_: 423).

As shown in Figure 3, Initial post-operative recovery was complicated by respiratory muscle weakness. On POD 2, the patient passed a spontaneous breathing trial (PaO_2_/FiO_2_ = 393) and was extubated. However, within 24 h, he experienced respiratory distress and desaturation (PaO_2_/FiO_2_ = 254), necessitating reintubation.

Over the next several days, the patient remained sedated with intermittent neuromuscular blockade (rocuronium and midazolam) due to ventilator–patient asynchrony. We performed bedside ultrasound, which showed reduced diaphragm excursion (< 10mm) and a thickening fraction of 17%, indicating dysfunction. A second extubation attempt on POD 5 again failed (PaO_2_/FiO_2_ = 259) while respiratory muscle weakness still persisted.

Structured respiratory rehabilitation was initiated by a multidisciplinary team starting from POD 6, including daily incentive spirometry, progressive limb mobilization, bedside physiotherapy and breathing coordination training. Oxygenation improved gradually, with PaO_2_/FiO_2_ increasing to 480 by POD 11. The third extubation on POD 12 was successful. We have clarified that the patient remained endotracheally intubated throughout the VV-ECMO course and confirmed that tracheostomy was considered but ultimately not performed.

Follow-up chest CT on POD 30 revealed improved graft aeration and resolution of pulmonary infiltrates. The patient was discharged in stable condition on POD 48 with satisfactory pulmonary function and oxygenation (SpO_2_ 96% on room air) and no signs of rejection (Figure 2B).

Because the paraquat ingestion was clearly suicidal, a psychiatry consultation was obtained shortly after transfer to our center. The psychiatric team diagnosed a depressive disorder with suicidal behavior, initiated supportive psychotherapy and pharmacologic treatment, and followed the patient regularly during the hospital stay. No recurrent suicidal ideation was documented prior to discharge, and outpatient psychiatric follow-up was arranged.

## Discussion

**Figure 4:**
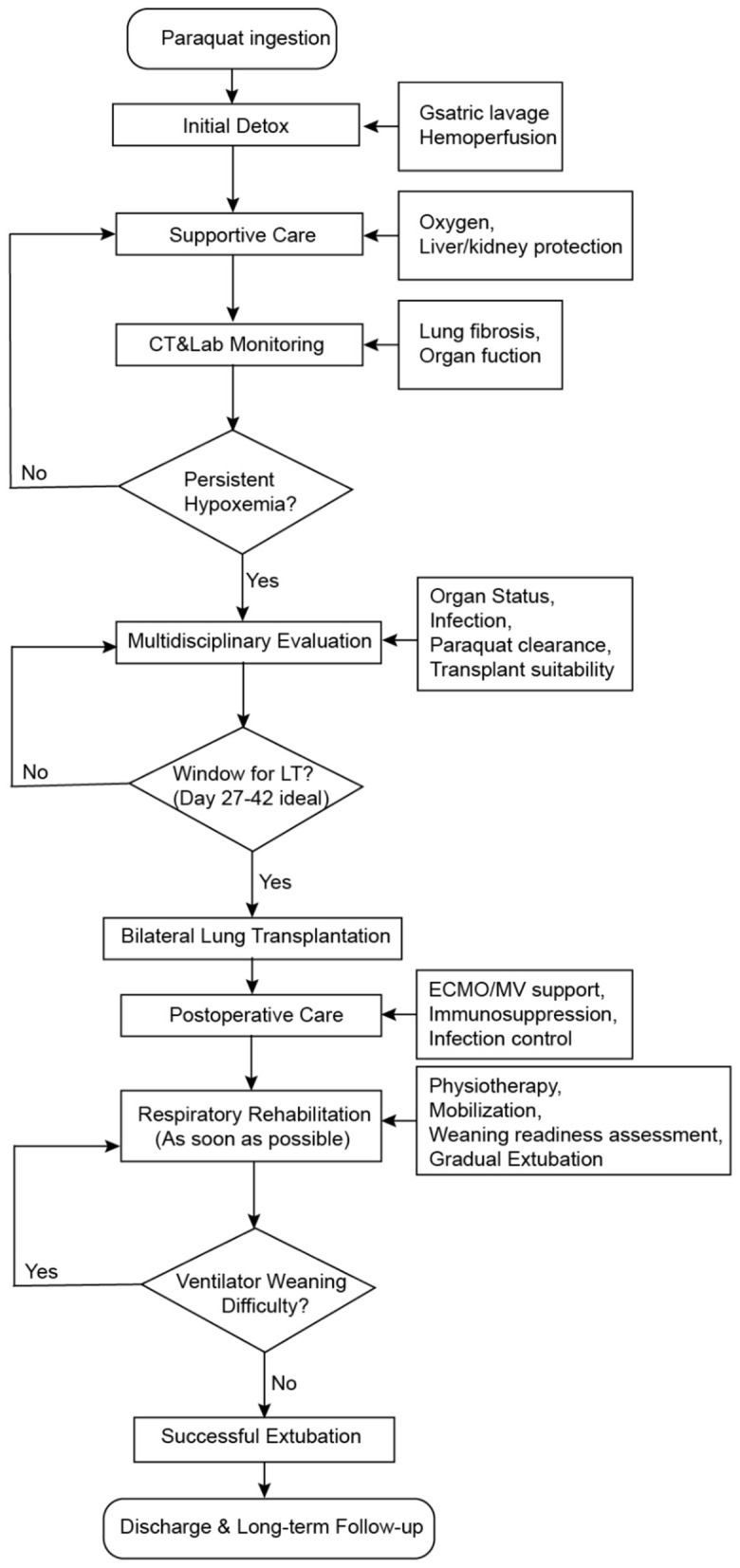
Proposed clinical management flowchart for paraquat-induced pulmonary fibrosis.

Lung transplantation (LT) for paraquat-induced acute pulmonary fibrosis is uniquely challenging due to the rapid progression of lung injury and multisystem involvement, necessitating meticulous perioperative management and individualized post-operative care (1). A key obstacle highlighted by our case was ventilator weaning failure resulting from prolonged sedation, neuromuscular blockade (NMBA) usage, diaphragmatic dysfunction, and ICU-acquired weakness (ICU-AW). Recent literature emphasizes that prolonged NMBAs and sedation independently predict ventilator-induced diaphragmatic dysfunction and ICU-AW, greatly complicating weaning (2). Early mobilization and structured rehabilitation protocols initiated within the first post-operative week significantly mitigate muscle atrophy and enhance weaning outcomes, as supported by randomized clinical trials in critically ill patients (3). However, such strategies are rarely detailed in published reports of paraquat-induced LT patients, highlighting our study's unique clinical contribution (Table 1). Based on our clinical experience and literature review, we developed a structured clinical decision-making flowchart to guide the management of patients with paraquat-induced acute lung injury undergoing lung transplantation. This algorithm may serve as a reference for centers managing similar toxic lung injury cases (Figure 4).

**Table 1:** Previous studies on post-operative mechanical ventilation in paraquat-induced lung fibrosis.

**Publication year**	**Transplant time after paraquat poisoning (d)**	**ECMO bridging (d)**	**Type of LT**	**Duration of mechanical ventilation (d)**	**Outcome**	**Poison concentration**
1968 (1)	6	/	Left Lung	NM	Died on the 16th day after poisoning	Lung: 8,500 ng/g
1973 (17)	10	NM	NM	NM	Death on respiratory failure	NM
1985 (18)	Right: 19 Left: 41	Right: 5 Left: 17	First right, then left	NM	Died on the 93 days after the initial LT because of massive tracheal hemorrhage	Blood: 0.26 μg/L After 2 days of left lung transplant: Blood: 0.2 μg/L; Rectus abdominis: 0.27 μg/L
1997 (19, 20)	44	NM	Left Lung	17	Survival	Lung: 134 μg/g, Muscle: 328 μg/g
2015 (6)	56	12	Bilateral	10	Survival	Blood: 30.53 ng/ml; Lung: 381.52 ng/g
2019 (2, 21–24)	55	23	Bilateral	14	Survival	NM
2019 (25)	NM	NM	Bilateral	NM	Survival	NM
2021 (26)	56	12	Bilateral	NM	Survival	Urine: 126.4 μg/ml
2021 (26)	38	/	Bilateral	NM	Survival	Urine: 229.2 μg/ml
2021 (24)	27	/	Bilateral	NM	Survival	Urine: 148.6 μg/ml
2021 (24)	27	1	Single	NM	Death on sepsis	Urine: 139.7 μg/ml
2021 (24)	28	/	Bilateral	NM	Survival	Urine: 126.4 μg/ml
2022 (25)	34	/	Bilateral	NM	Survival	Urine: 70 ng/mL
2023 (27)	47	35	Bilateral	NM	Survival	Urine: 22,900 μg/mL
2023 (28)	50	24	Bilateral	NM	Survival	NM

ECMO, Extracorporeal Membrane Oxygenation; NM, Not Mentioned; LT, Lung Transplant; d, days.

Timing of transplantation remains controversial (2). Early transplantation (< 2 weeks) historically demonstrated poor outcomes due to persistent paraquat tissue toxicity, while excessively delayed (>6 weeks) procedures risk irreversible multiorgan deterioration and infection (4, 5). Our patient's transplantation at day 34 aligns with recent successful cases, suggesting a pragmatic transplantation timeframe between 3 and 6 weeks post-ingestion, characterized by improved organ function and reduced systemic paraquat levels. Nonetheless, definitive biomarkers or objective criteria for transplantation timing remain elusive, underscoring a critical gap in the literature that future multicenter collaborations should address.

In addition to the physiological complexity of lung transplantation for paraquat-induced fibrosis, these patients often face substantial psychological challenges related to suicidal intent, underlying mood disorders, and the burden of long-term immunosuppressive therapy. Early and continuous involvement of a psychiatric team is therefore essential to address depression, support motivation for rehabilitation, and reduce the risk of recurrent suicidal behavior during and after the post-transplant recovery phase.

The role of ECMO bridging has evolved significantly, transforming prognoses for paraquat-induced lung injury patients (6). ECMO not only stabilizes severe hypoxemia but also supports perioperative hemodynamics, allowing safer transitions through transplantation (7). Emerging studies advocate early ECMO initiation (PaO_2_/FiO_2_ < 80–100) as a pivotal bridging strategy (8). Although prolonged ECMO increases infectious risks, brief perioperative ECMO use, as in our case, can optimize recovery outcomes.

In theory, early tracheostomy combined with an “awake” or ambulatory VV-ECMO strategy may confer important advantages. These benefits have been reported in selected ECMO populations bridged to lung transplantation, where awake ECMO has been associated with reduced immobilization and comparable post-transplant survival to conventionally managed patients (9). However, in the specific context of paraquat-related lung transplantation, the risk–benefit balance of tracheostomy is more complex. In the immediate post-transplant period, the bronchial anastomoses are particularly vulnerable to ischemia, infection, and mechanical trauma, and airway complications such as dehiscence, stenosis, granulation, and anastomotic infection occur in approximately 2–25% of recipients and are associated with considerable morbidity (10). Creating an additional airway stoma in this setting may theoretically increase the risks mentioned above. In our patient, given the anticipated short duration of post-operative ventilatory support, concern about early airway healing, and the observed rapid response to structured respiratory rehabilitation, we elected not to perform a tracheostomy. This case illustrates that while tracheostomy and ambulatory ECMO may be valuable options in selected long-bridge scenarios, their use in paraquat-related lung transplantation should be individualized, taking into account expected ECMO duration, airway status, infection risk, and rehabilitation needs.

Post-operative management for acute paraquat-induced fibrosis differs significantly from idiopathic pulmonary fibrosis (IPF), primarily due to the acute clinical trajectory and intensive preoperative treatments (ICU stay, ECMO bridging, sedation/NMBAs) involved. Paraquat patients typically demonstrate profound neuromuscular impairment and severe muscle weakness, mandating aggressive rehabilitation and neuromuscular recovery strategies immediately after transplantation (11). Conversely, IPF patients usually benefit from longer prehabilitation periods and elective transplantation settings, presenting fewer post-operative weaning challenges (12). Despite these differences, both patient groups require vigilant infection surveillance and tailored immunosuppressive regimens due to inherent post-operative infection risks and immunosuppressant-associated complications (13).

Similar to paraquat poisoning, other agents such as bleomycin, chlorine gas, and severe COVID-19 can lead to rapidly progressive pulmonary fibrosis requiring lung transplantation (14). These conditions share common clinical features, including refractory hypoxemia, prolonged mechanical ventilation, and poor response to anti-inflammatory therapies (15). However, the degree of systemic toxicity and multisystem involvement seen in paraquat poisoning is often more severe, demanding more aggressive perioperative support and individualized post-transplant rehabilitation strategies (16).

Our experience underscores a critical need to integrate structured respiratory rehabilitation, neuromuscular function monitoring, and proactive infection management early in post-operative care. This multidisciplinary approach-though described infrequently for paraquat-induced fibrosis patients-could significantly enhance clinical outcomes.

## Patient perspective

When I was first diagnosed with paraquat poisoning, I did not realize how serious my condition was. As my breathing became worse, I was transferred between hospitals and placed on machines that I did not fully understand. I remember feeling extremely weak, unable to speak, and uncertain whether I would survive. After being told that I would need a lung transplant to live, I was frightened but also relieved that there was still hope. The surgery and the days afterward were difficult. I could feel my body trying to recover, but it was hard to breathe on my own. I was frustrated when I couldn't be taken off the ventilator as planned, and I began to worry whether I would ever regain normal breathing.

The turning point came when the doctors and therapists started daily exercises and checked my muscle strength more closely. The rehabilitation team worked with me step by step, and I could feel my body gradually becoming stronger. Finally being able to breathe on my own again was a powerful moment I will never forget. Looking back, I am grateful not only for the transplant but for the patient care that focused on my recovery beyond surgery. This experience has taught me the value of perseverance and the importance of trusting the medical team.

## Conclusion

Paraquat poisoning is a rare but devastating cause of irreversible pulmonary fibrosis. Lung transplantation remains the only definitive treatment for select patients who survive the acute phase with preserved extrapulmonary function. In this case, we successfully performed bilateral lung transplantation under ECMO bridging. However, prolonged neuromuscular blockade, sedation, and systemic illness contributed to multiple weaning failures after surgery.

Our experience underscores the importance of implementing individualized and proactive rehabilitation strategies prior to extubation, particularly in patients with toxic lung injury and ICU-acquired weakness. Early physiotherapy, diaphragmatic training, and close neuromuscular assessment may significantly improve weaning success and long-term functional outcomes.

Furthermore, we constructed a structured clinical management flowchart that synthesizes lessons from this case and other published reports, integrating key decision points from poisoning onset to post-transplant recovery. This framework may not only help standardize care for paraquat-related lung injury but also serve as a reference for similar conditions such as chemical inhalation, drug-induced fibrosis, or post-ARDS fibrotic syndromes requiring lung transplantation.

## Data Availability

The raw data supporting the conclusions of this article will be made available by the authors, without undue reservation.

## Supplementary material

Supplementary Figure 1 Dynamics of paraquat elimination and organ function recovery.
