# Supplementary figures and images for: Case Report: Multifactorial weaning failure after lung transplantation in paraquat-induced pulmonary fibrosis: a case-based clinical review
Source: Front Med (Lausanne). 2026 Jan 21;12:1725363. doi: 10.3389/fmed.2025.1725363 (PMC12868150; doi:10.3389/fmed.2025.1725363)

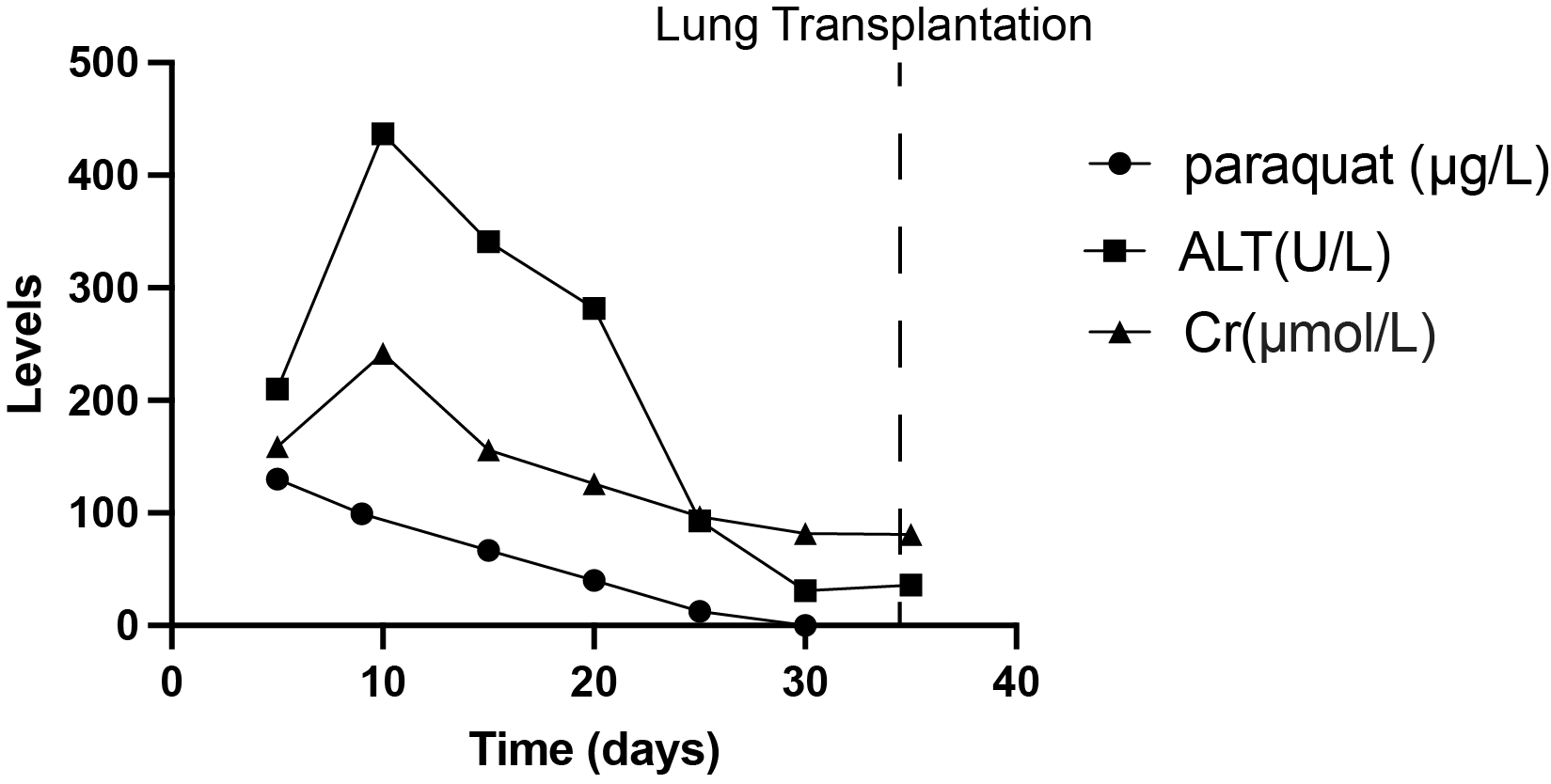

Supplement: Supplementary Figure 1 — Dynamics of paraquat elimination and organ function recovery. [file Image_1.tif]
